# Ethyl (*Z*)-4′-(4-eth­oxy-1-hy­droxy-3,4-dioxobut-1-en-1-yl)-[1,1′-biphen­yl]-3-carboxyl­ate

**DOI:** 10.1107/S1600536813018503

**Published:** 2013-07-10

**Authors:** Yoshinobu Ishikawa, Atsushi Ugai

**Affiliations:** a School of Pharmaceutical Sciences, University of Shizuoka, 52-1 Yada, Suruga-ku, Shizuoka 422-8526, Japan

## Abstract

The 1,3-diketone group of the title compound, C_21_H_20_O_6_, exists in a keto–enol form stabilized by a strong intra­molecular O—H⋯O hydrogen bond. As a result, a planar (mean deviation = 0.0099 Å) six-membered hydrogen-bonded ring is formed. The C—O and C—C bond lengths suggest significant electron delocalization in the ring. The dihedral angle between the six-membered hydrogen-bonded ring and its adjacent benzene ring is 8.78 (5)° and that between the benzene rings is 19.70 (5)°. In the crystal, mol­ecules are packed in a layered structure parallel to the *b* axis through C—H⋯O and π–π inter­actions [centroid–centroid distance between stacked benzene rings = 3.868 (2) Å].

## Related literature

For background to this study, see: Ishikawa & Fujii (2011 ▶). For related structures, see: Wang *et al.* (2008 ▶); Pillay *et al.* (2013 ▶). For the biological activity of related compounds, see: Tomassini *et al.* (1994 ▶).
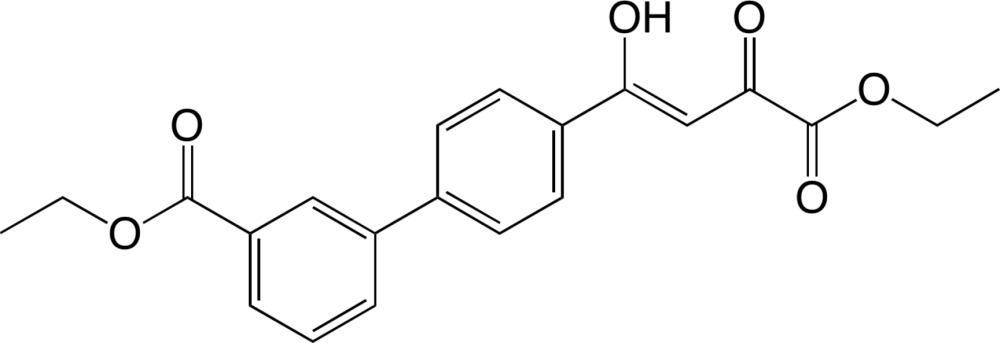


## Experimental

### 

#### Crystal data

C_21_H_20_O_6_
*M* *_r_* = 368.39
Monoclinic, 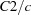

*a* = 26.572 (16) Å
*b* = 12.194 (5) Å
*c* = 11.213 (6) Å
β = 96.03 (5)°
*V* = 3613 (4) Å^3^
*Z* = 8
Mo *K*α radiation
μ = 0.10 mm^−1^
*T* = 100 K
0.50 × 0.45 × 0.30 mm

#### Data collection

Rigaku AFC-7R diffractometer
Absorption correction: ψ scan (North *et al.*, 1968 ▶) *T*_min_ = 0.707, *T*_max_ = 0.971
4944 measured reflections
4109 independent reflections
2874 reflections with *F* ^2^ > 2σ(*F* ^2^)
*R*_int_ = 0.029
3 standard reflections every 150 reflections intensity decay: 0.7%

#### Refinement

*R*[*F* ^2^ > 2σ(*F* ^2^)] = 0.040
*wR*(*F* ^2^) = 0.104
*S* = 1.01
4109 reflections
250 parameters
H atoms treated by a mixture of independent and constrained refinement
Δρ_max_ = 0.25 e Å^−3^
Δρ_min_ = −0.22 e Å^−3^

### 

Data collection: *WinAFC* (Rigaku, 1999 ▶); cell refinement: *WinAFC*; data reduction: *WinAFC*; program(s) used to solve structure: *SIR2008* (Burla *et al.*, 2007 ▶); program(s) used to refine structure: *SHELXL97* (Sheldrick, 2008 ▶); molecular graphics: *CrystalStructure* (Rigaku, 2010 ▶); software used to prepare material for publication: *CrystalStructure*.

## Supplementary Material

Crystal structure: contains datablock(s) General, I. DOI: 10.1107/S1600536813018503/ld2109sup1.cif

Structure factors: contains datablock(s) I. DOI: 10.1107/S1600536813018503/ld2109Isup2.hkl

Supplementary material file. DOI: 10.1107/S1600536813018503/ld2109Isup3.cml

Additional supplementary materials:  crystallographic information; 3D view; checkCIF report

## Figures and Tables

**Table 1:** Hydrogen-bond geometry (Å, °)

*D*—H⋯*A*	*D*—H	H⋯*A*	*D*⋯*A*	*D*—H⋯*A*
O3—H14⋯O4	0.98 (3)	1.61 (3)	2.522 (2)	152 (3)

**Fig. 1.**
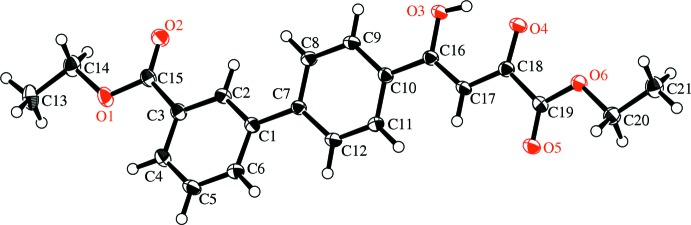
The molecular structure of the title compound, with displacement ellipsoids drawn at the 50% probability level. Hydrogen atoms are shown as small spheres of arbitrary radius.

**Fig. 2.**
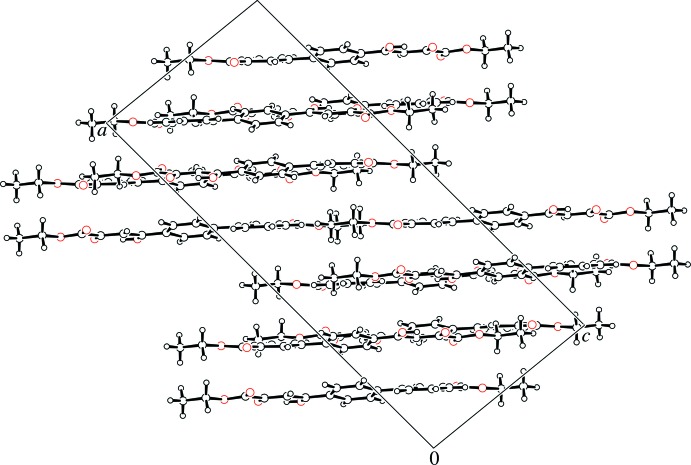
A crystal packing view of the title compound.

## supplementary crystallographic information

### Comment

Biphenyl diketo acids are known to inhibit influenza virus endonuclease (Tomassini *et al.* 1994). According to our inhibitor design targeting this metalloenzyme (Ishikawa *et al.* 2011), we synthesized the title compound by Claisen condensation of ethyl 4'-acetyl-[1,1'-biphenyl]-3-carboxylate with diethyl oxalate in the presence of sodium ethoxide, which biphenyl derivatives are the synthetic intermediates of a final biphenyl diketo acid. The 1,3-diketone group exists in keto-enol form stabilized by an intramolecular O–H···O hydrogen bond [O3···O4, 2.522 (2) Å, Table 1], as shown in Figure 1. The distances of C–O [C16–O3 = 1.3063 (18) Å, C18–O4 = 1.2736 (19) Å] and C–C bonds [C16–C17 = 1.403 (2) Å, C17–C18 = 1.396 (3) Å] in the 1,3-diketone group indicate charge delocalization among the atoms. As a result, a six-membered ring formed is essentially plane (mean deviation = 0.0099 Å). The dihedral angles between the six-membered ring and its adjacent benzene ring and between the two benzene rings are 8.78 (5) and 19.70 (5)°, respectively. In the crystal, the molecules are packed as a layered structure through intermolecular C–H···O and π···π interactions, as shown in Figure 2.

### Experimental

4'-Acetyl-[1,1'-biphenyl]-3-carboxylate (3.9 mmol), diethyl oxalate (4.7 mmol) and sodium ethoxide (5.4 mmol) were dissolved in ABS. THF (30 ml) and refluxed for 4 h. The reaction mixture was cooled to room temperature, and acidified with 2 M HCl (20 ml). After the mixture was extracted with ethyl acetate, the organic layer was washed with water and brine, and dried over anhydrous Mg_2_SO_4_. The title compound was obtained by column chromatography on silica gel (*n*-hexane: ethyl acetate = 3: 1, yield: 40%). Single crystals suitable for X-ray diffraction were obtained by slow evaporation of an ethyl acetate/*n*-hexane solution of the compound at room temperature.

### Refinement

The hydrogen atoms of phenyl ring were placed in geometrical positions [C–H 0.95 Å, *U*_iso_(H) = 1.2*U*_eq_(C)], and refined using a riding model. All hydrogen atoms of the vinyl, hydroxyl, methylene and methyl groups were found in a difference Fourier map, and refined using a riding model with distance constraint for the vinyl [C–H = 0.99 Å, *U*_iso_(H) = 1.2*U*_eq_(C)] and methylene H atoms [C–H = 0.95 Å, *U*_iso_(H) = 1.2*U*_eq_(C)]. A rotating group model was applied to the methyl groups with distance constraint [C–H = 0.98 Å, *U*_iso_(H) = 1.2*U*_eq_(C)]. The hydroxyl hydrogen was refined freely [O–H = 0.98 (3) Å].

### Crystal data

**Table:**

C_21_H_20_O_6_	*F*(000) = 1552.00
*M**_r_* = 368.39	*D*_x_ = 1.354 Mg m^−^^3^
Monoclinic, *C*2/*c*	Mo *K*α radiation, λ = 0.71069 Å
Hall symbol: -C 2yc	Cell parameters from 25 reflections
*a* = 26.572 (16) Å	θ = 15.3–17.3°
*b* = 12.194 (5) Å	µ = 0.10 mm^−^^1^
*c* = 11.213 (6) Å	*T* = 100 K
β = 96.03 (5)°	Block, yellow
*V* = 3613 (4) Å^3^	0.50 × 0.45 × 0.30 mm
*Z* = 8	

### Data collection

**Table:**

Rigaku AFC-7R diffractometer	*R*_int_ = 0.029
ω scans	θ_max_ = 27.5°
Absorption correction: ψ scan (North *et al.*, 1968)	*h* = −34→34
*T*_min_ = 0.707, *T*_max_ = 0.971	*k* = 0→15
4944 measured reflections	*l* = −14→8
4109 independent reflections	3 standard reflections every 150 reflections
2874 reflections with *F*^2^ > 2σ(*F*^2^)	intensity decay: 0.7%

### Refinement

**Table:**

Refinement on *F*^2^	Secondary atom site location: difference Fourier map
*R*[*F*^2^ > 2σ(*F*^2^)] = 0.040	Hydrogen site location: inferred from neighbouring sites
*wR*(*F*^2^) = 0.104	H atoms treated by a mixture of independent and constrained refinement
*S* = 1.01	*w* = 1/[σ^2^(*F*_o_^2^) + (0.0448*P*)^2^ + 1.6001*P*] where *P* = (*F*_o_^2^ + 2*F*_c_^2^)/3
4109 reflections	(Δ/σ)_max_ < 0.001
250 parameters	Δρ_max_ = 0.25 e Å^−^^3^
0 restraints	Δρ_min_ = −0.22 e Å^−^^3^
Primary atom site location: structure-invariant direct methods	

### Special details

**Table:**

Refinement. Refinement was performed using all reflections. The weighted *R*-factor (*wR*) and goodness of fit (*S*) are based on *F*^2^. *R*-factor (gt) are based on *F*. The threshold expression of *F*^2^ > 2.0 σ(*F*^2^) is used only for calculating *R*-factor (gt).

### Fractional atomic coordinates and isotropic or equivalent isotropic displacement parameters (Å^2^)

**Table:**

	*x*	*y*	*z*	*U*_iso_*/*U*_eq_	
O1	0.46554 (4)	0.31724 (9)	−0.39851 (9)	0.0275 (3)	
O2	0.42694 (4)	0.17877 (9)	−0.31120 (9)	0.0273 (3)	
O3	0.23852 (4)	0.18855 (8)	0.31124 (9)	0.0240 (3)	
O4	0.17956 (4)	0.23322 (8)	0.46659 (9)	0.0245 (3)	
O5	0.15319 (4)	0.51499 (8)	0.46408 (10)	0.0278 (3)	
O6	0.12949 (4)	0.38111 (8)	0.58408 (9)	0.0234 (3)	
C1	0.35694 (5)	0.41174 (11)	−0.10207 (12)	0.0193 (3)	
C2	0.37847 (5)	0.33352 (12)	−0.17183 (12)	0.0198 (3)	
C3	0.40986 (5)	0.36425 (12)	−0.25797 (12)	0.0212 (3)	
C4	0.41931 (6)	0.47473 (12)	−0.27835 (13)	0.0249 (4)	
C5	0.39742 (6)	0.55328 (12)	−0.21089 (14)	0.0260 (4)	
C6	0.36726 (5)	0.52305 (12)	−0.12270 (13)	0.0231 (3)	
C7	0.32531 (5)	0.37938 (11)	−0.00602 (12)	0.0183 (3)	
C8	0.32783 (5)	0.27412 (11)	0.04357 (13)	0.0218 (3)	
C9	0.29951 (5)	0.24567 (11)	0.13554 (13)	0.0221 (3)	
C10	0.26788 (5)	0.32256 (11)	0.18255 (12)	0.0194 (3)	
C11	0.26469 (5)	0.42780 (11)	0.13267 (13)	0.0216 (3)	
C12	0.29254 (5)	0.45500 (11)	0.03963 (13)	0.0219 (3)	
C13	0.51901 (6)	0.30330 (15)	−0.55382 (14)	0.0340 (4)	
C14	0.49283 (6)	0.23779 (13)	−0.46382 (14)	0.0291 (4)	
C15	0.43394 (5)	0.27607 (12)	−0.32420 (12)	0.0213 (3)	
C16	0.23830 (5)	0.29209 (11)	0.28132 (12)	0.0195 (3)	
C17	0.21060 (5)	0.36805 (11)	0.34202 (12)	0.0201 (3)	
C18	0.18231 (5)	0.33257 (12)	0.43284 (13)	0.0202 (3)	
C19	0.15335 (5)	0.42051 (11)	0.49450 (12)	0.0203 (3)	
C20	0.10152 (6)	0.46341 (12)	0.64510 (14)	0.0265 (4)	
C21	0.07504 (6)	0.40539 (14)	0.73898 (14)	0.0297 (4)	
H1	0.3716	0.2580	−0.1604	0.0238*	
H2	0.4405	0.4959	−0.3376	0.0299*	
H3	0.4031	0.6288	−0.2253	0.0312*	
H4	0.3534	0.5780	−0.0757	0.0277*	
H5	0.2894	0.5264	0.0059	0.0263*	
H6	0.2432	0.4809	0.1630	0.0259*	
H7	0.3016	0.1733	0.1670	0.0265*	
H8	0.3494	0.2210	0.0135	0.0262*	
H9C	0.5393	0.2540	−0.5986	0.0408*	
H10B	0.5411	0.3582	−0.5116	0.0408*	
H11A	0.4936	0.3401	−0.6096	0.0408*	
H12A	0.4691	0.1840	−0.5052	0.0350*	
H13B	0.5179	0.1978	−0.4085	0.0350*	
H14	0.2155 (10)	0.182 (3)	0.374 (3)	0.093 (9)*	
H15	0.2111	0.4436	0.3214	0.0242*	
H16B	0.0765	0.5005	0.5870	0.0318*	
H17A	0.1250	0.5194	0.6829	0.0318*	
H18A	0.0509	0.3523	0.7003	0.0356*	
H19B	0.0569	0.4591	0.7835	0.0356*	
H20C	0.1000	0.3669	0.7944	0.0356*	

### Atomic displacement parameters (Å^2^)

**Table:**

	*U*^11^	*U*^22^	*U*^33^	*U*^12^	*U*^13^	*U*^23^
O1	0.0321 (6)	0.0274 (6)	0.0253 (6)	−0.0054 (5)	0.0144 (5)	−0.0032 (5)
O2	0.0332 (6)	0.0216 (6)	0.0288 (6)	−0.0009 (5)	0.0111 (5)	−0.0018 (5)
O3	0.0289 (6)	0.0146 (5)	0.0300 (6)	0.0008 (5)	0.0104 (5)	0.0027 (5)
O4	0.0312 (6)	0.0165 (5)	0.0272 (6)	−0.0002 (4)	0.0094 (5)	0.0034 (4)
O5	0.0335 (6)	0.0162 (6)	0.0359 (6)	−0.0007 (5)	0.0139 (5)	0.0015 (5)
O6	0.0283 (6)	0.0197 (5)	0.0237 (6)	0.0009 (5)	0.0098 (5)	−0.0004 (4)
C1	0.0192 (7)	0.0195 (7)	0.0191 (7)	−0.0011 (6)	0.0023 (6)	0.0012 (6)
C2	0.0226 (7)	0.0175 (7)	0.0194 (7)	−0.0020 (6)	0.0024 (6)	0.0002 (6)
C3	0.0224 (7)	0.0227 (8)	0.0188 (7)	−0.0020 (6)	0.0031 (6)	−0.0001 (6)
C4	0.0262 (8)	0.0257 (8)	0.0237 (7)	−0.0037 (6)	0.0071 (6)	0.0031 (6)
C5	0.0312 (8)	0.0173 (7)	0.0304 (8)	−0.0027 (6)	0.0068 (7)	0.0036 (6)
C6	0.0255 (8)	0.0179 (7)	0.0262 (8)	0.0006 (6)	0.0045 (6)	−0.0002 (6)
C7	0.0204 (7)	0.0156 (7)	0.0190 (7)	−0.0014 (6)	0.0027 (6)	−0.0004 (6)
C8	0.0258 (8)	0.0157 (7)	0.0249 (8)	0.0021 (6)	0.0071 (6)	−0.0011 (6)
C9	0.0264 (8)	0.0145 (7)	0.0259 (8)	0.0000 (6)	0.0050 (6)	0.0017 (6)
C10	0.0201 (7)	0.0171 (7)	0.0211 (7)	−0.0022 (6)	0.0033 (6)	−0.0004 (6)
C11	0.0229 (7)	0.0166 (7)	0.0260 (8)	0.0014 (6)	0.0056 (6)	−0.0008 (6)
C12	0.0258 (8)	0.0157 (7)	0.0247 (8)	0.0010 (6)	0.0048 (6)	0.0028 (6)
C13	0.0285 (9)	0.0509 (11)	0.0240 (8)	−0.0059 (8)	0.0092 (7)	−0.0023 (8)
C14	0.0311 (9)	0.0330 (9)	0.0248 (8)	−0.0032 (7)	0.0101 (7)	−0.0076 (7)
C15	0.0226 (7)	0.0236 (8)	0.0179 (7)	−0.0040 (6)	0.0030 (6)	−0.0006 (6)
C16	0.0197 (7)	0.0167 (7)	0.0221 (7)	−0.0023 (6)	0.0019 (6)	0.0006 (6)
C17	0.0236 (7)	0.0143 (7)	0.0234 (7)	−0.0018 (6)	0.0065 (6)	0.0022 (6)
C18	0.0203 (7)	0.0189 (7)	0.0213 (7)	−0.0009 (6)	0.0014 (6)	0.0003 (6)
C19	0.0205 (7)	0.0197 (7)	0.0206 (7)	−0.0030 (6)	0.0023 (6)	0.0008 (6)
C20	0.0306 (8)	0.0222 (8)	0.0282 (8)	0.0010 (6)	0.0105 (7)	−0.0041 (6)
C21	0.0311 (9)	0.0333 (9)	0.0256 (8)	−0.0030 (7)	0.0070 (7)	−0.0047 (7)

### Geometric parameters (Å, º)

**Table:**

O1—C14	1.453 (2)	C16—C17	1.403 (2)
O1—C15	1.3410 (19)	C17—C18	1.396 (3)
O2—C15	1.2122 (19)	C18—C19	1.527 (2)
O3—C16	1.3063 (18)	C20—C21	1.503 (3)
O4—C18	1.2736 (19)	O3—H14	0.98 (3)
O5—C19	1.2014 (18)	C2—H1	0.950
O6—C19	1.3325 (19)	C4—H2	0.950
O6—C20	1.461 (2)	C5—H3	0.950
C1—C2	1.394 (2)	C6—H4	0.950
C1—C6	1.409 (2)	C8—H8	0.950
C1—C7	1.487 (3)	C9—H7	0.950
C2—C3	1.392 (3)	C11—H6	0.950
C3—C4	1.394 (3)	C12—H5	0.950
C3—C15	1.489 (2)	C13—H9C	0.980
C4—C5	1.386 (3)	C13—H10B	0.980
C5—C6	1.387 (3)	C13—H11A	0.980
C7—C8	1.398 (2)	C14—H12A	0.990
C7—C12	1.401 (2)	C14—H13B	0.990
C8—C9	1.383 (3)	C17—H15	0.950
C9—C10	1.399 (2)	C20—H16B	0.990
C10—C11	1.399 (2)	C20—H17A	0.990
C10—C16	1.471 (3)	C21—H18A	0.980
C11—C12	1.381 (3)	C21—H19B	0.980
C13—C14	1.513 (3)	C21—H20C	0.980
			
O1···C4	2.713 (2)	C13···H2^viii^	2.9857
O2···C2	2.843 (2)	C13···H3^viii^	3.4897
O2···C14	2.672 (3)	C13···H3^vii^	3.5519
O3···O4	2.522 (2)	C13···H18A^ii^	3.0176
O3···C9	2.768 (3)	C13···H18A^ix^	3.5234
O3···C18	2.757 (2)	C13···H20C^ix^	3.5510
O4···O5	3.506 (2)	C14···H18A^ii^	2.9897
O4···O6	2.6695 (18)	C15···H13B^i^	2.9416
O4···C16	2.819 (3)	C16···H1^ii^	3.1478
O5···C17	2.802 (2)	C16···H5^vi^	3.5161
O5···C20	2.643 (3)	C17···H1^ii^	3.2167
C1···C4	2.816 (3)	C17···H5^vi^	2.9340
C2···C5	2.770 (3)	C17···H17A^vii^	3.0634
C2···C8	2.975 (3)	C18···H1^ii^	3.4183
C3···C6	2.774 (3)	C18···H5^vi^	3.3520
C6···C12	2.950 (3)	C18···H14^iv^	3.30 (3)
C7···C10	2.821 (3)	C18···H17A^vii^	3.5401
C8···C11	2.771 (3)	C19···H6^vi^	3.1253
C9···C12	2.769 (3)	C19···H12A^ii^	3.5092
C11···C17	2.969 (3)	C19···H17A^vii^	3.5716
O1···C15^i^	3.496 (3)	C20···H7^iv^	3.5648
O2···O4^ii^	3.341 (3)	C20···H12A^ii^	2.9325
O2···O5^ii^	3.505 (2)	C20···H15^vi^	3.5299
O2···O6^ii^	3.344 (3)	C21···H3^iii^	3.4284
O2···C18^ii^	3.078 (3)	C21···H7^iv^	3.4721
O2···C19^ii^	3.053 (3)	C21···H8^iv^	3.5970
O2···C20^iii^	3.352 (3)	C21···H9C^x^	2.8914
O2···C21^iii^	3.432 (3)	C21···H12A^ii^	2.9651
O3···O4^iv^	3.273 (3)	C21···H18A^xi^	3.5436
O3···C1^ii^	3.490 (3)	C21···H19B^xi^	3.5504
O3···C2^ii^	3.340 (3)	H1···O3^ii^	3.2877
O3···C11^iii^	3.244 (2)	H1···O4^ii^	3.5590
O3···C12^iii^	3.446 (2)	H1···C16^ii^	3.1478
O3···C18^iv^	3.385 (3)	H1···C17^ii^	3.2167
O4···O2^ii^	3.341 (3)	H1···C18^ii^	3.4183
O4···O3^iv^	3.273 (3)	H1···H9C^i^	3.5497
O4···C2^ii^	3.591 (3)	H1···H10B^i^	3.1063
O4···C3^ii^	3.371 (3)	H1···H13B^i^	3.0453
O4···C6^iii^	3.411 (2)	H1···H14^ii^	3.2330
O4···C12^iii^	3.475 (3)	H1···H16B^iii^	3.4926
O4···C15^ii^	3.263 (3)	H1···H17A^iii^	2.9232
O4···C16^iv^	3.399 (3)	H2···C6^vii^	3.5703
O5···O2^ii^	3.505 (2)	H2···C13^viii^	2.9857
O5···C8^v^	3.202 (2)	H2···H2^i^	3.5455
O5···C9^v^	3.322 (2)	H2···H4^vii^	3.4624
O5···C11^vi^	3.415 (3)	H2···H9C^viii^	3.1920
O5···C21^vii^	3.244 (3)	H2···H10B^viii^	2.5369
O6···O2^ii^	3.344 (3)	H2···H11A^viii^	2.7640
C1···O3^ii^	3.490 (3)	H2···H11A^vi^	3.4310
C2···O3^ii^	3.340 (3)	H3···C7^vii^	3.5820
C2···O4^ii^	3.591 (3)	H3···C8^vii^	3.3219
C2···C13^i^	3.537 (3)	H3···C9^vii^	3.3851
C2···C16^ii^	3.561 (3)	H3···C13^viii^	3.4897
C3···O4^ii^	3.371 (3)	H3···C13^vi^	3.5519
C6···O4^v^	3.411 (2)	H3···C21^v^	3.4284
C8···O5^iii^	3.202 (2)	H3···H9C^viii^	2.9870
C8···C10^ii^	3.596 (3)	H3···H10B^viii^	3.4412
C9···O5^iii^	3.322 (2)	H3···H11A^viii^	3.4889
C10···C8^ii^	3.596 (3)	H3···H11A^vi^	2.6355
C11···O3^v^	3.244 (2)	H3···H18A^v^	2.9882
C11···O5^vii^	3.415 (3)	H3···H20C^v^	3.0038
C12···O3^v^	3.446 (2)	H4···O4^v^	2.4644
C12···O4^v^	3.475 (3)	H4···C10^vii^	3.5624
C13···C2^i^	3.537 (3)	H4···H2^vi^	3.4624
C15···O1^i^	3.496 (3)	H4···H14^v^	3.3091
C15···O4^ii^	3.263 (3)	H5···O3^v^	2.9972
C15···C18^ii^	3.463 (3)	H5···O4^v^	2.6608
C16···O4^iv^	3.399 (3)	H5···C16^vii^	3.5161
C16···C2^ii^	3.561 (3)	H5···C17^vii^	2.9340
C18···O2^ii^	3.078 (3)	H5···C18^vii^	3.3520
C18···O3^iv^	3.385 (3)	H5···H14^v^	2.3365
C18···C15^ii^	3.463 (3)	H5···H15^vii^	2.8002
C19···O2^ii^	3.053 (3)	H6···O3^v^	2.5881
C20···O2^v^	3.352 (3)	H6···O5^vii^	3.0949
C21···O2^v^	3.432 (3)	H6···O6^vii^	3.4902
C21···O5^vi^	3.244 (3)	H6···C19^vii^	3.1253
O1···H2	2.3982	H6···H7^v^	3.3205
O1···H9C	3.2233	H6···H14^v^	2.7309
O1···H10B	2.5354	H6···H17A^vii^	3.1715
O1···H11A	2.5700	H7···O5^iii^	2.7758
O2···H1	2.5449	H7···O6^iv^	3.2419
O2···H12A	2.5507	H7···C12^ii^	3.5853
O2···H13B	2.7648	H7···C20^iv^	3.5648
O3···H7	2.4545	H7···C21^iv^	3.4721
O3···H15	3.1988	H7···H6^iii^	3.3205
O4···H14	1.61 (3)	H7···H15^iii^	2.8263
O4···H15	3.1969	H7···H17A^iv^	3.3859
O5···H15	2.4907	H7···H20C^iv^	2.6507
O5···H16B	2.5829	H8···O5^iii^	2.5269
O5···H17A	2.6402	H8···C21^iv^	3.5970
O6···H18A	2.5990	H8···H9C^i^	3.0378
O6···H19B	3.2448	H8···H10B^i^	3.3570
O6···H20C	2.5671	H8···H16B^iii^	3.5833
C1···H3	3.2829	H8···H17A^iii^	3.4169
C1···H5	2.6641	H8···H20C^iv^	2.6434
C1···H8	2.6798	H9C···C8^i^	3.5293
C2···H2	3.2759	H9C···C21^ix^	2.8914
C2···H4	3.2622	H9C···H1^i^	3.5497
C2···H8	2.6707	H9C···H2^viii^	3.1920
C3···H3	3.2539	H9C···H3^viii^	2.9870
C4···H1	3.2700	H9C···H8^i^	3.0378
C4···H4	3.2636	H9C···H11A^xii^	3.4602
C6···H1	3.2632	H9C···H18A^ii^	2.8535
C6···H2	3.2687	H9C···H18A^ix^	2.6475
C6···H5	2.6431	H9C···H19B^ix^	2.9744
C7···H1	2.6736	H9C···H20C^ix^	2.5743
C7···H4	2.6748	H10B···C1^i^	2.9426
C7···H6	3.2793	H10B···C2^i^	2.8215
C7···H7	3.2765	H10B···C3^i^	3.1638
C8···H1	2.6799	H10B···C4^viii^	3.3647
C8···H5	3.2553	H10B···C6^i^	3.3854
C9···H6	3.2652	H10B···C7^i^	3.5428
C10···H5	3.2666	H10B···H1^i^	3.1063
C10···H8	3.2685	H10B···H2^viii^	2.5369
C10···H14	3.18 (3)	H10B···H3^viii^	3.4412
C10···H15	2.7149	H10B···H8^i^	3.3570
C11···H7	3.2650	H11A···C4^viii^	3.5559
C11···H15	2.6786	H11A···C4^vii^	3.4333
C12···H4	2.6412	H11A···C5^vii^	2.9830
C12···H8	3.2562	H11A···H2^viii^	2.7640
C15···H1	2.6085	H11A···H2^vii^	3.4310
C15···H2	2.6912	H11A···H3^viii^	3.4889
C15···H12A	2.5793	H11A···H3^vii^	2.6355
C15···H13B	2.6884	H11A···H9C^xii^	3.4602
C16···H6	2.6678	H11A···H11A^xii^	3.2029
C16···H7	2.6493	H11A···H18A^ii^	2.7724
C17···H6	2.6534	H12A···O6^ii^	2.7919
C17···H14	2.30 (3)	H12A···C19^ii^	3.5092
C18···H14	2.17 (3)	H12A···C20^ii^	2.9325
C19···H15	2.6130	H12A···C21^ii^	2.9651
C19···H16B	2.5769	H12A···H16B^ii^	2.6725
C19···H17A	2.6111	H12A···H18A^ii^	2.2402
H1···H8	2.1435	H12A···H19B^ii^	3.5794
H2···H3	2.3368	H13B···O2^i^	2.7405
H3···H4	2.3256	H13B···C2^i^	3.2567
H4···H5	2.1096	H13B···C3^i^	3.2453
H5···H6	2.3181	H13B···C15^i^	2.9416
H6···H15	2.0993	H13B···H1^i^	3.0453
H7···H8	2.3193	H13B···H16B^xiii^	2.8690
H7···H14	3.4244	H14···O4^iv^	3.32 (3)
H9C···H12A	2.3887	H14···C2^ii^	3.19 (3)
H9C···H13B	2.3637	H14···C3^ii^	3.49 (3)
H10B···H12A	2.8649	H14···C11^iii^	3.14 (3)
H10B···H13B	2.3859	H14···C12^iii^	2.94 (3)
H11A···H12A	2.3613	H14···C18^iv^	3.30 (3)
H11A···H13B	2.8648	H14···H1^ii^	3.2330
H14···H15	3.2475	H14···H4^iii^	3.3091
H16B···H18A	2.3503	H14···H5^iii^	2.3365
H16B···H19B	2.3716	H14···H6^iii^	2.7309
H16B···H20C	2.8545	H15···C12^vi^	3.3303
H17A···H18A	2.8545	H15···C20^vii^	3.5299
H17A···H19B	2.3484	H15···H5^vi^	2.8002
H17A···H20C	2.3735	H15···H7^v^	2.8263
O2···H13B^i^	2.7405	H15···H17A^vii^	2.6640
O2···H16B^iii^	3.3321	H16B···O2^v^	3.3321
O2···H17A^iii^	2.8585	H16B···H1^v^	3.4926
O2···H19B^iii^	2.7254	H16B···H8^v^	3.5833
O3···H1^ii^	3.2877	H16B···H12A^ii^	2.6725
O3···H5^iii^	2.9972	H16B···H13B^xiv^	2.8690
O3···H6^iii^	2.5881	H16B···H19B^vii^	3.4230
O4···H1^ii^	3.5590	H17A···O2^v^	2.8585
O4···H4^iii^	2.4644	H17A···O5^vi^	3.1902
O4···H5^iii^	2.6608	H17A···C17^vi^	3.0634
O4···H14^iv^	3.32 (3)	H17A···C18^vi^	3.5401
O5···H6^vi^	3.0949	H17A···C19^vi^	3.5716
O5···H7^v^	2.7758	H17A···H1^v^	2.9232
O5···H8^v^	2.5269	H17A···H6^vi^	3.1715
O5···H17A^vii^	3.1902	H17A···H7^iv^	3.3859
O5···H19B^vii^	3.1066	H17A···H8^v^	3.4169
O5···H20C^vii^	2.6696	H17A···H15^vi^	2.6640
O6···H6^vi^	3.4902	H18A···C13^ii^	3.0176
O6···H7^iv^	3.2419	H18A···C13^x^	3.5234
O6···H12A^ii^	2.7919	H18A···C14^ii^	2.9897
C1···H10B^i^	2.9426	H18A···C21^xi^	3.5436
C2···H10B^i^	2.8215	H18A···H3^iii^	2.9882
C2···H13B^i^	3.2567	H18A···H9C^ii^	2.8535
C2···H14^ii^	3.19 (3)	H18A···H9C^x^	2.6475
C3···H10B^i^	3.1638	H18A···H11A^ii^	2.7724
C3···H13B^i^	3.2453	H18A···H12A^ii^	2.2402
C3···H14^ii^	3.49 (3)	H18A···H18A^xi^	3.0315
C4···H10B^viii^	3.3647	H18A···H19B^xi^	3.1694
C4···H11A^viii^	3.5559	H19B···O2^v^	2.7254
C4···H11A^vi^	3.4333	H19B···O5^vi^	3.1066
C5···H11A^vi^	2.9830	H19B···C21^xi^	3.5504
C6···H2^vi^	3.5703	H19B···H9C^x^	2.9744
C6···H10B^i^	3.3854	H19B···H12A^ii^	3.5794
C7···H3^vi^	3.5820	H19B···H16B^vi^	3.4230
C7···H10B^i^	3.5428	H19B···H18A^xi^	3.1694
C8···H3^vi^	3.3219	H19B···H19B^xi^	3.0404
C8···H9C^i^	3.5293	H20C···O5^vi^	2.6696
C8···H20C^iv^	3.0334	H20C···C8^iv^	3.0334
C9···H3^vi^	3.3851	H20C···C9^iv^	3.0310
C9···H20C^iv^	3.0310	H20C···C13^x^	3.5510
C10···H4^vi^	3.5624	H20C···H3^iii^	3.0038
C11···H14^v^	3.14 (3)	H20C···H7^iv^	2.6507
C12···H7^ii^	3.5853	H20C···H8^iv^	2.6434
C12···H14^v^	2.94 (3)	H20C···H9C^x^	2.5743
C12···H15^vii^	3.3303		
			
C14—O1—C15	116.21 (13)	C3—C2—H1	119.466
C19—O6—C20	114.27 (12)	C3—C4—H2	120.476
C2—C1—C6	117.95 (14)	C5—C4—H2	120.481
C2—C1—C7	121.42 (13)	C4—C5—H3	119.592
C6—C1—C7	120.60 (13)	C6—C5—H3	119.564
C1—C2—C3	121.08 (14)	C1—C6—H4	119.650
C2—C3—C4	120.35 (14)	C5—C6—H4	119.663
C2—C3—C15	118.15 (14)	C7—C8—H8	119.343
C4—C3—C15	121.47 (14)	C9—C8—H8	119.332
C3—C4—C5	119.04 (15)	C8—C9—H7	119.710
C4—C5—C6	120.84 (14)	C10—C9—H7	119.736
C1—C6—C5	120.69 (14)	C10—C11—H6	119.748
C1—C7—C8	121.64 (13)	C12—C11—H6	119.758
C1—C7—C12	120.66 (13)	C7—C12—H5	119.317
C8—C7—C12	117.68 (14)	C11—C12—H5	119.315
C7—C8—C9	121.33 (13)	C14—C13—H9C	109.471
C8—C9—C10	120.55 (13)	C14—C13—H10B	109.472
C9—C10—C11	118.55 (14)	C14—C13—H11A	109.458
C9—C10—C16	120.26 (13)	H9C—C13—H10B	109.476
C11—C10—C16	121.19 (13)	H9C—C13—H11A	109.476
C10—C11—C12	120.49 (13)	H10B—C13—H11A	109.474
C7—C12—C11	121.37 (13)	O1—C14—H12A	110.568
O1—C14—C13	105.84 (14)	O1—C14—H13B	110.569
O1—C15—O2	123.72 (14)	C13—C14—H12A	110.576
O1—C15—C3	111.68 (13)	C13—C14—H13B	110.560
O2—C15—C3	124.58 (14)	H12A—C14—H13B	108.723
O3—C16—C10	116.68 (12)	C16—C17—H15	120.025
O3—C16—C17	120.13 (13)	C18—C17—H15	120.024
C10—C16—C17	123.19 (13)	O6—C20—H16B	110.178
C16—C17—C18	119.95 (13)	O6—C20—H17A	110.180
O4—C18—C17	124.30 (14)	C21—C20—H16B	110.180
O4—C18—C19	119.08 (13)	C21—C20—H17A	110.180
C17—C18—C19	116.62 (13)	H16B—C20—H17A	108.472
O5—C19—O6	124.89 (14)	C20—C21—H18A	109.474
O5—C19—C18	122.13 (14)	C20—C21—H19B	109.479
O6—C19—C18	112.98 (12)	C20—C21—H20C	109.475
O6—C20—C21	107.65 (13)	H18A—C21—H19B	109.468
C16—O3—H14	106.3 (16)	H18A—C21—H20C	109.466
C1—C2—H1	119.454	H19B—C21—H20C	109.466
			
C14—O1—C15—O2	−1.11 (18)	C12—C7—C8—H8	179.2
C14—O1—C15—C3	177.38 (10)	C7—C8—C9—C10	−0.80 (19)
C15—O1—C14—C13	171.47 (10)	C7—C8—C9—H7	179.2
C15—O1—C14—H12A	51.7	H8—C8—C9—C10	179.2
C15—O1—C14—H13B	−68.8	H8—C8—C9—H7	−0.8
H14—O3—C16—C10	−176.2 (15)	C8—C9—C10—C11	1.45 (19)
H14—O3—C16—C17	3.5 (15)	C8—C9—C10—C16	−179.30 (11)
C19—O6—C20—C21	177.39 (10)	H7—C9—C10—C11	−178.6
C19—O6—C20—H16B	57.2	H7—C9—C10—C16	0.7
C19—O6—C20—H17A	−62.4	C9—C10—C11—C12	−0.48 (19)
C20—O6—C19—O5	0.51 (18)	C9—C10—C11—H6	179.5
C20—O6—C19—C18	179.85 (10)	C9—C10—C16—O3	−8.07 (18)
C2—C1—C6—C5	0.80 (18)	C9—C10—C16—C17	172.22 (11)
C2—C1—C6—H4	−179.2	C11—C10—C16—O3	171.16 (11)
C6—C1—C2—C3	1.00 (18)	C11—C10—C16—C17	−8.55 (19)
C6—C1—C2—H1	−179.0	C16—C10—C11—C12	−179.72 (11)
C2—C1—C7—C8	19.70 (18)	C16—C10—C11—H6	0.3
C2—C1—C7—C12	−161.64 (11)	C10—C11—C12—C7	−1.16 (19)
C7—C1—C2—C3	−177.12 (11)	C10—C11—C12—H5	178.8
C7—C1—C2—H1	2.9	H6—C11—C12—C7	178.8
C6—C1—C7—C8	−158.37 (11)	H6—C11—C12—H5	−1.2
C6—C1—C7—C12	20.29 (18)	H9C—C13—C14—O1	177.7
C7—C1—C6—C5	178.94 (11)	H9C—C13—C14—H12A	−62.5
C7—C1—C6—H4	−1.1	H9C—C13—C14—H13B	58.0
C1—C2—C3—C4	−1.72 (19)	H10B—C13—C14—O1	57.7
C1—C2—C3—C15	176.49 (11)	H10B—C13—C14—H12A	177.5
H1—C2—C3—C4	178.3	H10B—C13—C14—H13B	−62.0
H1—C2—C3—C15	−3.5	H11A—C13—C14—O1	−62.3
C2—C3—C4—C5	0.6 (2)	H11A—C13—C14—H12A	57.5
C2—C3—C4—H2	−179.4	H11A—C13—C14—H13B	178.0
C2—C3—C15—O1	−176.05 (11)	O3—C16—C17—C18	−1.40 (18)
C2—C3—C15—O2	2.42 (19)	O3—C16—C17—H15	178.6
C4—C3—C15—O1	2.14 (17)	C10—C16—C17—C18	178.29 (11)
C4—C3—C15—O2	−179.39 (12)	C10—C16—C17—H15	−1.7
C15—C3—C4—C5	−177.53 (11)	C16—C17—C18—O4	0.2 (2)
C15—C3—C4—H2	2.5	C16—C17—C18—C19	179.90 (10)
C3—C4—C5—C6	1.2 (2)	H15—C17—C18—O4	−179.8
C3—C4—C5—H3	−178.8	H15—C17—C18—C19	−0.1
H2—C4—C5—C6	−178.8	O4—C18—C19—O5	−177.60 (11)
H2—C4—C5—H3	1.2	O4—C18—C19—O6	3.04 (17)
C4—C5—C6—C1	−1.9 (2)	C17—C18—C19—O5	2.66 (18)
C4—C5—C6—H4	178.1	C17—C18—C19—O6	−176.70 (11)
H3—C5—C6—C1	178.1	O6—C20—C21—H18A	−62.1
H3—C5—C6—H4	−1.9	O6—C20—C21—H19B	177.9
C1—C7—C8—C9	177.89 (11)	O6—C20—C21—H20C	57.9
C1—C7—C8—H8	−2.1	H16B—C20—C21—H18A	58.1
C1—C7—C12—C11	−176.92 (11)	H16B—C20—C21—H19B	−61.9
C1—C7—C12—H5	3.1	H16B—C20—C21—H20C	178.1
C8—C7—C12—C11	1.78 (19)	H17A—C20—C21—H18A	177.7
C8—C7—C12—H5	−178.2	H17A—C20—C21—H19B	57.7
C12—C7—C8—C9	−0.80 (19)	H17A—C20—C21—H20C	−62.3

Symmetry codes: (i) −*x*+1, *y*, −*z*−1/2; (ii) −*x*+1/2, −*y*+1/2, −*z*; (iii) −*x*+1/2, *y*−1/2, −*z*+1/2; (iv) −*x*+1/2, −*y*+1/2, −*z*+1; (v) −*x*+1/2, *y*+1/2, −*z*+1/2; (vi) *x*, −*y*+1, *z*+1/2; (vii) *x*, −*y*+1, *z*−1/2; (viii) −*x*+1, −*y*+1, −*z*−1; (ix) *x*+1/2, −*y*+1/2, *z*−3/2; (x) *x*−1/2, −*y*+1/2, *z*+3/2; (xi) −*x*, *y*, −*z*+3/2; (xii) −*x*+1, *y*, −*z*−3/2; (xiii) *x*+1/2, *y*−1/2, *z*−1; (xiv) *x*−1/2, *y*+1/2, *z*+1.

### Hydrogen-bond geometry (Å, º)

**Table:**

*D*—H···*A*	*D*—H	H···*A*	*D*···*A*	*D*—H···*A*
O3—H14···O4	0.98 (3)	1.61 (3)	2.522 (2)	152 (3)
